# Supplementary material for: Orthologs at the Base of the Olfactores Clade
Source: Genes (Basel). 2024 May 22;15(6):657. doi: 10.3390/genes15060657 (PMC11203038; doi:10.3390/genes15060657)
Supplement: Supplementary file 1 [file genes-15-00657-s001.zip › genes-2986109-supplementary.pdf]

SUPPLEMENTARY MATERIAL

Table S1 Tunicate orthologs of vertebrate proteins

Tunicate gene Unique ID*	HGNC symbol	Uniprot	Specific role	Expect value**	Description taken from GeneCards database (see Methods)	Selected References
Phmamm.g00013747	ADGRL3	Q9HAR2		0 as Latrophilin 3 of *Phallusia mammilata*	Plays a role in cell-cell adhesion and neuron guidance .	[56]
Cirobu.g00013347	AES aka TLES	Q08117		4.00E-59	similar in sequence to theDrosophila enhancer of split groucho, a protein involved in neurogenesis during embryonic development.	
Cirobu.g00012489	AKAP2	Q9Y2D5		4.00E-25	May be involved in establishing polarity in signaling systems	
Phmamm.g00004896	ASXL3	Q9C0F0		2E-43 - as *Phallusia mammillata* SXL3 precursor	act by forming multiprotein complexes, which are required to maintain the transcriptionally repressive state of homeotic genes throughout development.	
Phmamm.g00004987	CALD1	Q05682		5E-38 as non-muscle caldesmon of *Phallusia mamillata*	plays an essential role in the regulation of smooth muscle and nonmuscle contraction.	[57]
Cirobu.g00003956	CBLN1	P23435		9.00E-39	connecting synapses	
Cirobu.g00002666	CDH1	P12830		1E-115 as Ciona cadherin isoform X1	Involved in mechanisms regulating cell-cell adhesions, mobility and proliferation of epithelial cells	[58]
Cirobu.g00010294	CDH2	P19022		5.00E-12	may be involved in neuronal recognition mechanism, may regulate dendritic spine density and control neural tube closure (GeneCards)	[59]
Cirobu.g00002666	CDH7	Q9ULB5		2.00E-147	cadherin 7	
Coinfl.g00007259	CDH8	P55286		7E-152 as Ciona Type II cadherin	expressed in brain and is putatively involved in synaptic adhesion,	[58]

				axon outgrowth and guidance .	
Cisavi.g00011235	CDH11	P55287	2E-109 as *Ciona intestinalis* cadherin precursor	its upregulation during differentiation, suggests a specific function in bone development and maintenance .	[60]
Cirobu.g00001398	CDH16	O75309	3E-40 as *Ciona savigny* Type II cadherin	may play a role in the morphological organization of liver and intestine .	
Cirobu.g00002666	CDH18	Q13634	2E-146 as Ciona Type II cadherin	This particular cadherin is expressed specifically in the central nervous system and is putatively involved in synaptic adhesion, axon outgrowth and guidance .	[58]
Cirobu.g00009056	CLDN1	O95832	8.00E-42	Tight junctions	[28]
Cirobu.g00002206	CLDN18	P56856	1.00E-29	Claudin18.Claudins are integral membrane proteins and components of tight junction strands, serving as a physical barrier between epithelial or endothelial cell sheets .	
Cirobu.g00002476	CLDN19	Q8N6F1	2.00E-40	Tight junctions	[28]
Cirobu.g00012793	CLDND1	Q9NY35	4.00E-21	Located in cell surface.	[61]
Cirobu.g00014646	COL22A1	Q8NFW1	0	Acts as a cell adhesion ligand for skin epithelial cells and fibroblasts	
Boleac.g00009169	COL23A1	Q86Y22	5.00E-113	contains a single pass hydrophobic transmembrane domain .	[62]
Cirobu.g00014791	CRYGN	Q8WXF5	6E-17 vs gamma crystallin S (aka Cirobu.g00014791) of Ciona intestinalis	Crystallins are the dominant structural components of the vertebrate eye lens .	[46]
Cirobu.g00014791	CRYGS	P22914	E-16 vs gamma crystallin S (aka Cirobu.g00014791) of Ciona intestinalis	Crystallins are the dominant structural components of the vertebrate eye lens .	[46]

Cirobu.g00008072	DLX2	Q07687	3E-29 as DLX-b of Oikopleura dioica	DLX gene family contain a homeobox that is related to that of Distal-less (Dll), a gene expressed in the head and limbs of the developing embryo	[63]
Cirobu.g00005203	DLX3	O60479	2 E-35 as DLX-c of Oikopleura dioica	Mutations in this gene have been associated with the autosomal dominant conditions trichodentoosseous syndrome.	[63]
Cirobu.g00005364	EFNA1	P20827	4E-15 - as Phallus mammillata A-a precursor	crucial for migration, repulsion and adhesion during neuronal, vascular and epithelial development.	[64]
Cirobu.g00015386	EFNA2	O43921	1E-19 - as Phallus mammillata A-b precursor	crucial for migration, repulsion and adhesion during neuronal, vascular and epithelial development.	[30]
Cirobu.g00005966	EFNA3	P52797	4E-17 - as Phallus mammillata A-c precursor	Among its related pathways are Nervous system development and EPH-Ephrin signaling.	[64]
Cirobu.g00005966	EFNA4	P52798	1E-20 - as Phallus mammillata A-d precursor	implicated in mediating developmental events, especially in the nervous system and in erythropoiesis.	[30]
Cirobu.g00005364	EFNA5	P52803	3E-21 - as *Ciona intestinalis* precursor	crucial for migration, repulsion and adhesion during neuronal, vascular and epithelial development.	
Cirobu.g00013004	FAM3D	Q96BQ1	2.00E-58	regulation of glucose metabolism	[65]
Cirobu.g00013155	FBXO24	O75426	2.00E-76	involved in ubiquitinylation.	[66]
Cirobu.g00012273	FGF12	P61328	5E-63 as *Botryllus schlosseri* fibroblast growth factor 11/12/13/14	FGF family members possess broad mitogenic and cell survival activities, and are involved in a variety of biological	[67]

					processes, including embryonic development, cell growth, morphogenesis, tissue repair, tumor growth and invasion .	
Cirobu.g00014816	FGF5	P12034		1E-31 as Ciona intestinalis fibroblast growth factor 4/5/6	FGF family members possess broad mitogenic and cell survival activities, and are involved in a variety of biological processes, including embryonic development, cell growth, morphogenesis, tissue repair, tumor growth and invasion .	[67]
Cisavi.g00003564	FLRT2	O43155		3.00E-62	regulates early embryonic vascular and neural development .	[68]
Cirobu.g00007675	FLRT3	Q9NZU0		2E-54 as *Ciona intestinalis* FLRT	Functions in cell-cell adhesion, cell migration and axon guidance .	[68]
Cirobu.g00013271	FZD3	Q9NPG1		2.00E-147	May be involved in transduction and intercellular transmission of polarity information during tissue morphogenesis (CeneCards)	
Cirobu.g00013271	FZD6	O60353		3E-134 vs PhallusiaFZ3/6	May be involved in transduction and intercellular transmission of polarity information during tissue morphogenesis (CeneCards)	
Cirobu.g00015669	GJA4	P35212	Gap Junction gene	4.00E-41	Gap Junction Protein Alpha 4	
Cirobu.g00002251	GJA5	P36382	Gap Junction gene	1.00E-32	Gap Junction Protein Alpha 5	
Cirobu.g00004266	GJA8	P48165	Gap Junction gene	3.00E-57	Gap Junction Protein Alpha 8	

Cirobu.g00007788	GJB1	P08034	Gap Junction gene	6.00E-34	Gap Junction Protein Beta 1	
Cirobu.g00007721	GJB2	P29033	Gap Junction gene	7.00E-35	Gap Junction Protein Beta 2	
Cirobu.g00014910	GJB6	O95452	Gap Junction gene	3.00E-47	Gap Junction Protein Beta 6	
Cirobu.g00006387	GJC1	P36383	Gap Junction gene	3.00E-67	Gap Junction Protein Gamma 1	
Harore.g00000543	GNRH1	P01148		6E-8 as Halocynthia roretzi hormone	Acts on the brain, retina, sympathetic nervous system, gonads and placenta in certain species.	[69]
Cirobu.g00014915	H1FOO	Q8IZA3		9.00E-27	(From Aniseed) pseudogene no summaries	
Cirobu.g00014541	HSPB1	P04792		1.00E-46	When overexpressed in C.elegans HSBP1 has severe effects on survival of the animals after thermal and chemical stress	
Cirobu.g00005867	IRX5	P78411		4E-28 as IRX-e of *Oikpleuro dioica*	Involved in craniofacial and gonadal development .	[70]
Cirobu.g00007177	MAGED1	Q9Y5V3		7.00E-42	May enhance ubiquitin ligase activity of RING-type zinc finger-containing E3 ubiquitin-protein ligases .	[37]
Cirobu.g00007177	MAGED2	Q9UNF1		1.00E-47	thought to play a role in cell cycle regulation and response to stress	[37]
Cirobu.g00007177	MAGED3	Q12816		2.00E-41	aka TRO trophinin - mediates cell adhesion between trophoblastic cells and the epithelial cells of the endometrium .	[37]
Cirobu.g00007177	MAGED4	Q96JG8		4.00E-41	May enhance ubiquitin ligase activity of RING-type zinc finger-containing E3 ubiquitin-protein ligases .	[37]
Boschl.g00017367	MIA	Q16674		1.00E-14	involved in extracellular matrix organization .	

Cirobu.g00006549	MYADML2	A6NDP7		3.00E-12	elements of the machinery that controls specialized pathways of membrane traffic and cell signaling	[71]
Cirobu.g00000146	MYBPC1	Q00872	Muscle-related gene	0	plays an important role in muscle contraction by recruiting muscle-type creatine kinase to myosin filaments.	
Cirobu.g00000146	MYBPC2	Q14324	Muscle-related gene	0	The protein encoded by this locus is referred to as the fast-type isoform .	
Cirobu.g00000146	MYBPC3	Q14896	Muscle-related gene	0	heart muscle actin-binding	[72]
Boschl.g00071919	MYH1	P12882	Muscle-related gene	0 as myosin heavy chain, cardiac muscle isoform *Ciona intestinalis*	Myosin is a major contractile protein which converts chemical energy into mechanical energy through the hydrolysis of ATP.	[73]
Harore.g00014293	MYH13	Q9UKX3	Muscle-related gene	0 as embryonic muscle myosin heavy chain *Halocynthia roretzi*	Predicted to enable microfilament motor activity	[73]
Harore.g00003537	MYH15	Q9Y2K3	Muscle-related gene	0 as embryonic muscle myosin heavy chain *Halocynthia roretzi*	Predicted to be involved in extraocular skeletal muscle development .	[73]
Cirobu.g00005587	MYH4	Q9Y623	Muscle-related gene	0 as myosin heavy chain, cardiac muscle isoform *Ciona intestinalis*	Located in myofibril.	[73]
Harore.g00010698	MYH8	P13535	Muscle-related gene	0 as embryonic muscle myosin heavy chain *Halocynthia roretzi*	This gene is predominantly expressed in fetal skeletal muscle .	[73]
Cirobu.g00008856	MYL4	P12829	Muscle-related gene	2E-74 as smooth muscle isoform X3 of Ciona intestinalis	encodes a myosin alkali light chain that is found in embryonic muscle and adult atria .	[74]
Cirobu.g00008931	MYL5	Q02045	Muscle-related gene	2.00E-82	mitotic spindle	[75]
Cirobu.g00009534	MYL7	Q01449	Muscle-related gene	3E-73 as smooth muscle isoform X1 of Ciona intestinalis	predicted to enable calcium ion binding activity .	[76]

Phmamm.g00001992	MYOM1	P52179	Muscle-related gene	7.00E-93	Binds myosin, titin, and light meromyosin .	[77]
Cirobu.g00001822	NECTIN1	Q15223		6.00E-24	an adhesion protein that plays a role in the organization of adherens junctions and tight junctions in epithelial and endothelial cells .	
Cirobu.g00001822	NECTIN3	Q9NQS3		6.00E-31	precursor of adaptive immunity system	[78]
Cirobu.g00008036	PALLD	Q8WX93	Muscle-related gene	1.00E-109	Cytoskeletal protein required for organization of normal actin cytoskeleton. Roles in establishing cell morphology, motility, cell adhesion and cell-extracellular matrix interactions	[79]
Cirobu.g00011526	PITX3	O75364		2E-63 as pituitary homeobox pitx isoform a/b [Ciona intestinalis]	involved in lens formation during eye development .	[80]
Cisavi.g00003397	PCDH12	Q9NPG4		7E-83 as protocadherin *Ciona savignyi*	Acts as a regulator of cell migration, probably via increasing cell-cell adhesion .	[22]
Cisavi.g00009430	PCDHGB7	Q9Y5F8		5E-89 as protocadherin *Ciona savignyi*	neural cadherin-like cell adhesion protein most likely playing a critical role in the establishment and function of specific cell-cell connections in the brain.	
Cisavi.g00002522	RGS18	Q9NS28		4.00E-27	Inhibits signal transduction by increasing the GTPase activity of G protein alpha subunits thereby driving them into their inactive GDP-bound form.	
Boleac.g00006277	SERPINA4	P29622		3.00E-41	Predicted to enable serine-type endopeptidase inhibitor activity	
Cirobu.g00003628	STXBP6	Q8NFX7		3.00E-38	As amisyn_Role in the SNARE conplex . in synapse vesicle fusion	[81]

Boleac.g00003200	SYNPO2	Q9UMS6	Muscle-related gene	2.00E-13	Involved in positive regulation of actin filament bundle assembly; positive regulation of cell migration .	
Cirobu.g00008275	TFAP2B	Q92481		1E-124 vs generic TFAP2	involved in a large spectrum of important biological functions including proper eye, face, body wall, limb and neural tube development	
Cirobu.g00000301	TFAP2E	Q6VUC0		6E-109 vs generic TFAP2	involved in a large spectrum of important biological functions including proper eye, face, body wall, limb and neural tube development	
Cirobu.g00003009	TMEM150B	A6NC51		8.00E-19	May have some role in extracellular matrix engulfment or growth factor receptor recycling, both of which can modulate cell survival .	
Cirobu.g00007201	TMPRSS11A	Q6ZMR5		2.00E-44	may play a role in cellular senescence. Overexpression inhibits cell growth and induce G1 cell cycle arrest .	
Phfumi.g00001551	TMPRSS12	Q86WS5		2.00E-58	Required for normal sperm motility and binding to the zona pellucida .	
Cirobu.g00006671	TNNT1	P13805	Muscle-related gene	8E-85 as TroponinT, slow skletal muscle of *Ciona intestinalis*	This protein is the slow skeletal troponin T subunit.	[82]
Cirobu.g00012836	ZIC3	O60481		5.00E-63	functions as a transcription factor in early stages of left-right body axis formation in higher animals	[83]
Cirobu.g00014064+A45	ZMAT1	Q5H9K5		2.00E-17	This gene encodes a protein containing(C2H2)-type zinc fingers, which are similar to those found	

in the nuclear matrix protein matrin 3 .

TABLE S2. DAVID ANALYSIS OF TUNICATE NON-CODING GENES (Functional Annotation Table)

ID	GOTERM_Biological Process_DIRECT
LINC01783	GO:0006412~translation,
*MIR124-2**	GO:0035195~gene silencing by miRNA,
*MIR124-3**	GO:0035195~gene silencing by miRNA,
MIR133B	GO:0010831~+ve regulation of myotube differentiation, and, GO:0010976~ of neuron development, and of GO:1905563~ vascular endothelial cell proliferation.
MIR141	GO:0008284~positive regulation of cell proliferation
MIR153-1	GO:1903817~negative regulation of voltage-gated potassium channel activity,GO:1905651~regulation of artery morphogenesis.
MIR153-2	GO:0016442~RISC complex,
MIR155HG	GO:0035195~gene silencing by miRNA,
MIR183	GO:0010812~negative regulation of cell-substrate adhesion,GO:0090263~positive regulation of canonical Wnt signaling pathway,
MIR216A	GO:0035195~gene silencing by miRNA,
MIR216B	GO:0035195~gene silencing by miRNA,
MIR217	GO:0016525~negative regulation of angiogenesis.
MIR219A1	GO:0010977~negative regulation of neuron projection development.
MIR25	GO:0055022~negative regulation of cardiac muscle tissue growth,GO:1903243~negative regulation of cardiac muscle hypertrophy in response to stress.
MIR31	,GO:0090050~positive regulation of cell migration involved in sprouting angiogenesis,GO:1903672~positive regulation of sprouting angiogenesis.
MIR33A	GO:0010629~negative regulation of gene expression
MIR33B	GO:0035195~gene silencing by miRNA.
*MIR7-2***	GO:0035195~gene silencing by miRNA,
*MIR7-3***	GO:0035195~gene silencing by miRNA,
MIRLET7A2	GO:0035195~gene silencing by miRNA,
MIRLET7C	GO:0035195~gene silencing by miRNA, GO:1904893~negative regulation of STAT cascade.
MIRLET7G	GO:0030336~negative regulation of cell migration.

Footnote - Genes in the above Table shown directly to be expressed in the Ascidian *Ciona intestinalis* include:

**MIR124-2* and *MIR124-3*, required for nervous system development [84]

****MIR7-2* and *MIR7-3*, expressed in the peripheral nervous system [85].

Supplementary Figure S1

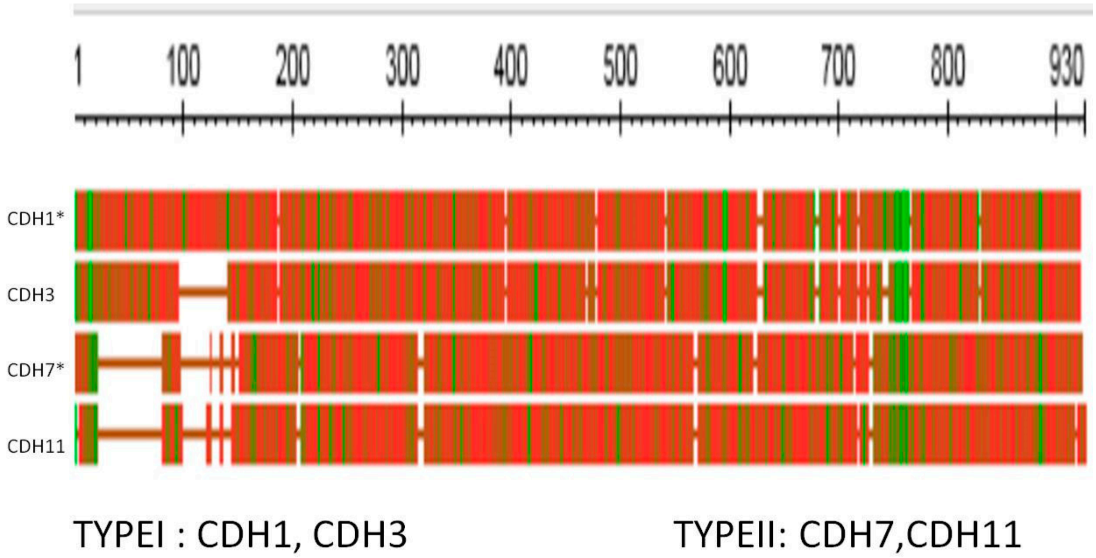

Legend to Supplementary Figure S1: The upper two rows depict cadherins of type I; the lower rows depict those of type II. In each case an ortholog found in the tunicates is marked with an asterisk.

Supplementary Figure S2:

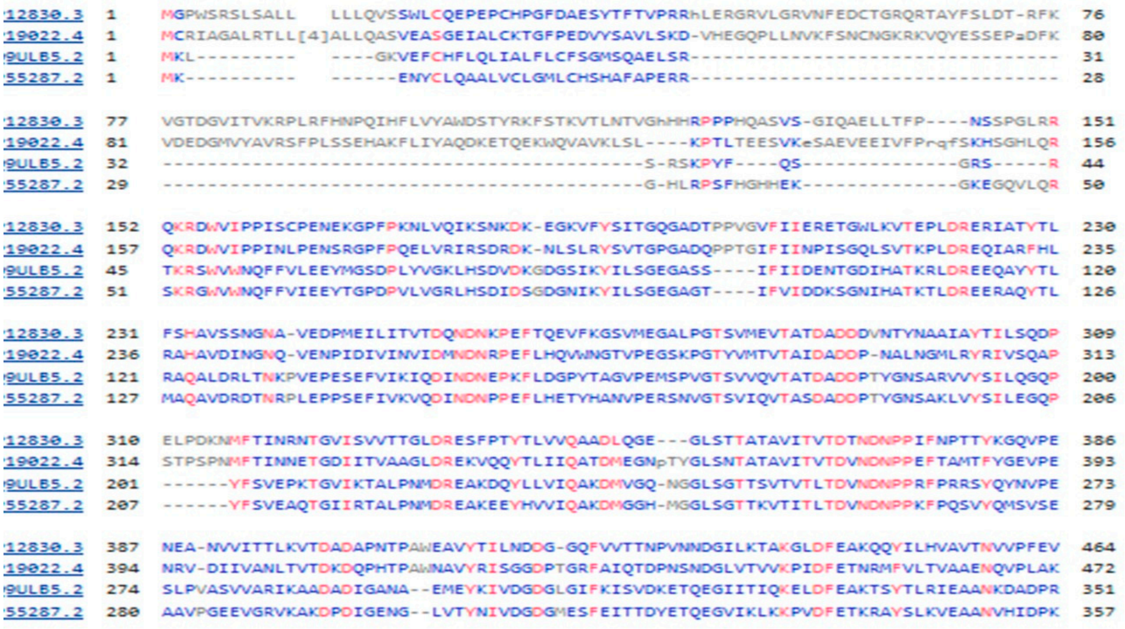

Legend to Supplementary Figure S2**.** Expanded first section of Supplementary Figure S2. The upper two rows depict cadherins of type I; the lower rows depict those of type II

Supplementary Figure S3

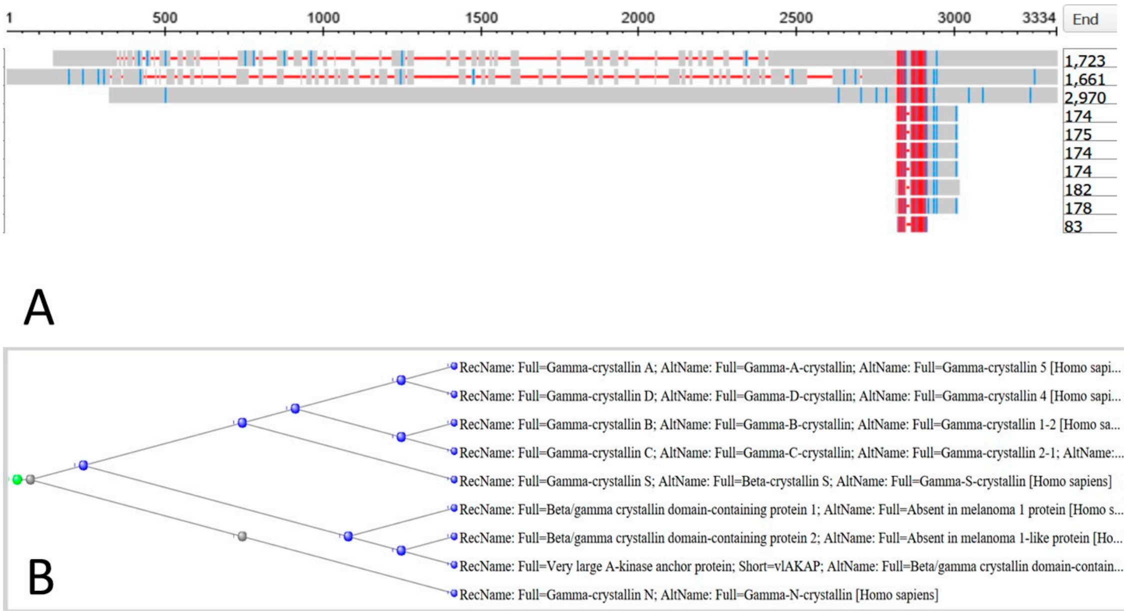

Legend to Supplementary Figure S3: Part A depicts a COBALT-based alignment of the gamma crystallin proteins (top three rows) compared with the six gamma crystallins (next six) and the single tunicate crystalline (short lowest sequence). Part B is a phylogram from these data showing their separation into two families.
